# Complete plastid genome of *Cumathamnion serrulatum* (Ceramiales, Rhodophyta)

**DOI:** 10.1080/23802359.2021.1920489

**Published:** 2021-06-15

**Authors:** Hocheol Kim, Do-Yun Lee, Chang Wan Seo, Chung Hyun Cho, Hwan Su Yoon

**Affiliations:** a Department of Biological Sciences, Sungkyunkwan University, Suwon, Korea; b College of Pharmacy, Seoul National University, Seoul, Korea; c School of Biological Sciences, Seoul National University, Seoul, Korea

**Keywords:** Plastid genome, red algae, Ceramiales

## Abstract

We report the complete plastid genome of *Cumathamnion serrulatum,* also known as *Delesseria serrulata*. The plastid genome was 174,192 bp in size. Annotation showed there were 193 protein coding genes, three ribosomal RNAs, and 29 transfer RNAs. One intron was found, and the GC content was 27.2%. The maximum likelihood tree with the concatenated 177 plastid coding genes showed a strong monophyletic relationship to *Membranoptera* spp. within the Ceramiales.

*Cumathamnion serrulatum* (Harvey) M.J. Wynne & G.W. Saunders 2012, previously known as *Delesseria serrulata,* is a red algal species of the Ceramiales, Florideophyceae, Rhodophyta (Wynne and Saunders 2012). *Cumathamnion serrulatum* is widely distributed in Korea (Choi et al. 2002), Japan (Yoshida et al. 1990), Russia (Kozhenkova 2009), and North America (Wynne and Saunders 2012). The thallus is bright rosy-red in color and its primary frond produces branches irregularly from the midrib. Numerous secondary fronds emerge with similar shapes, followed by third and fourth leaflets in a similar way. All leaflets are linear-lanceolate, but they vary in degree of attenuation (Wynne and Saunders 2012). Despite its ecological and pharmacological importance (Güven et al. 2010; Kim et al. 2012), no genomic study has been reported yet. Here, we report the complete plastid genome of *C. serrulatum*.

*Cumathamnion serrulatum* was collected in Gonghyeonjin, Gangwon-do, Korea (38°21'29.1"N 128°30'37.7"E) on 28 March 2015. A voucher specimen was deposited at Sungkyunkwan University Herbarium (https://www.skku.edu, HSY, hsyoon2011@skku.edu) under accession number SKKU003380. Before DNA extraction from a leaflet of thallus, epiphytic contaminants were removed with brushes, forceps and sterile water. Genomic DNA was extracted using LaboPass™ Tissue Genomic DNA Isolation Kit Mini (COSMO Genetech, Seoul, Korea) following the manufacturer’s instructions. Total DNA was cleaned up again to remove impurities using DNeasy PowerClean Cleanup Kit (QIAGEN, Hilden, Germany). Sequencing libraries were prepared using an Ion Xpress Plus gDNA Fragment Library Preparation kit for the 400 bp libraries (Thermo Fisher Scientific, San Francisco, California, USA). Sequencing reads were generated by an Ion Personal Genome Machine (PGM) platform using the Ion PGM Hi-Q Template kit and the Ion PGM Hi-Q sequencing kit (Thermo Fisher Scientific, San Francisco, California, USA).

Raw reads were trimmed and assembled with CLC Genomics Workbench 5.5.1 (CLC Bio, Aarhus, Denmark) and MIRA3 Assembler (Chevreux et al. 1999). Plastid contigs were sorted out using tBLASTn. A consensus sequence was confirmed with a read-mapping tool in CLC Genomics Workbench to correct sequencing errors and fill gaps. Protein coding genes were manually annotated with the aid of NCBI’s BLASTx function. Then, these genes were confirmed with published red algal plastid genomes (see Figure 1). The rRNA sequences were identified using the RNAmmer 1.2 Server with the option of bacteria (Lagesen et al. 2007) and re-confirmed using BLASTn on NCBI. The tRNAs were checked using the ARAGORN web server (Laslett and Canback 2004). Lastly, ambiguous regions were re-sequenced using PCR amplification followed by Sanger sequencing of amplicons. The newly assembled plastid genome of *C. serrulatum* (GenBank accession number: MW292565) was 174,192 bp in size. The plastid genome contained 193 protein coding genes (CDSs), three rRNAs, 29 tRNAs, and one intron. GC content was 27.2%.

**Figure 1.**
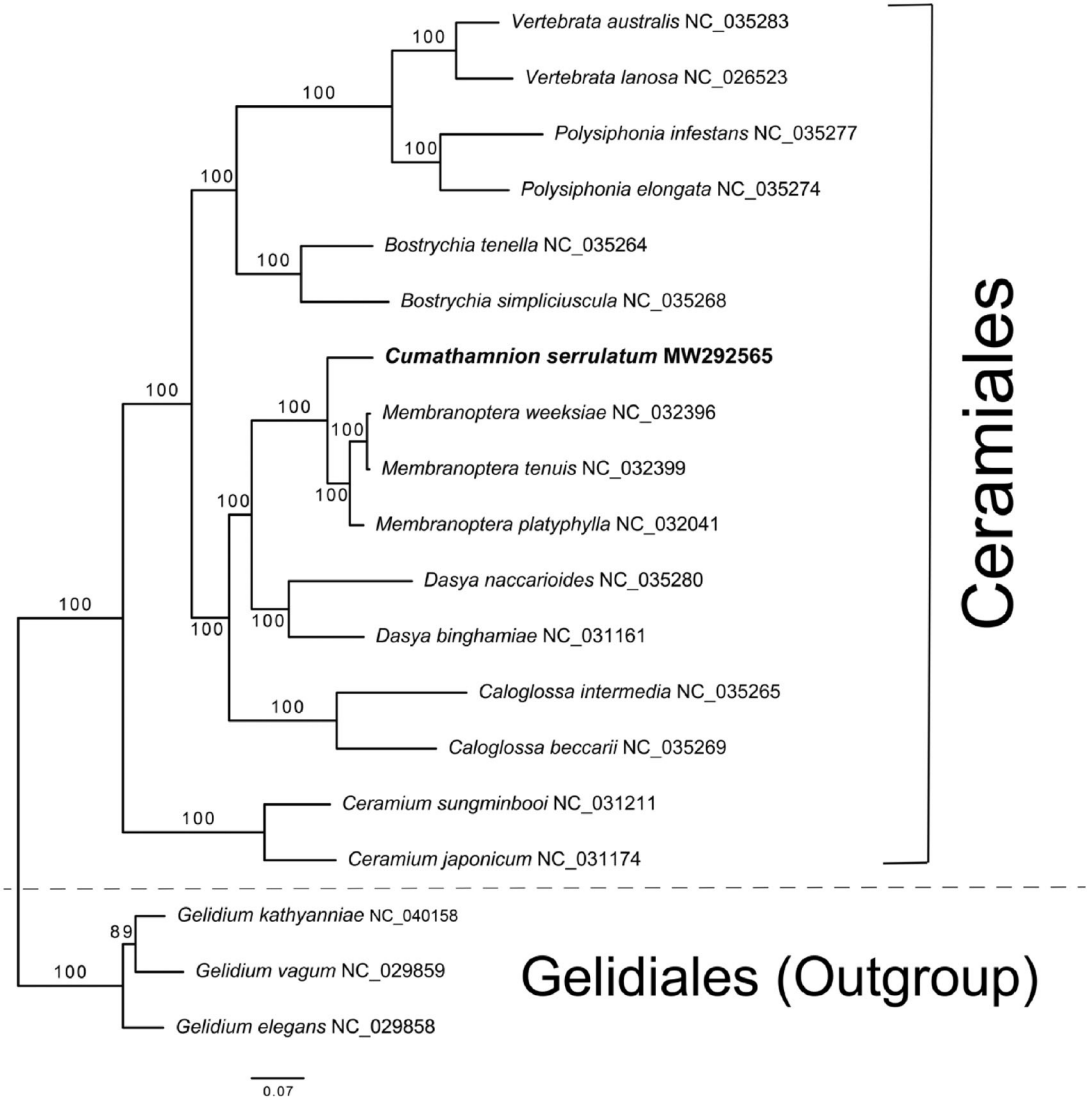
Maximum likelihood (ML) tree with 177 concatenated plastid proteins from 16 species of Ceramiales and three species of Gelidales (Outgroup).

To construct a phylogeny with the concatenated plastid CDSs and to infer the phylogenetic position of *C. serrulatum*, 15 available plastid genomes of the Ceramiales were selected with an outgroup of three Gelidales species (Salomaki et al. 2015; Lee, Cho, et al. 2016a; Lee, Kim et al. 2016b; Hughey and Boo 2016; Tamayo and Hughey 2016; Díaz-Tapia et al. 2017; Hughey et al. 2017; Boo and Hughey 2019). The extraction of CDSs from plastid genomes was conducted following a method described in Lee et al. (Lee, Cho, et al. 2016a; Lee, Kim, et al. 2016b). A total of 177 gene-sets were aligned by MAFFT v7.310 (Katoh and Standley 2013). A maximum likelihood (ML) tree was constructed by IQ-tree v1.6.12 with 1000 ultrafast bootstrap replicates (Nguyen et al. 2015). The phylogeny showed a strong monophyletic relationship of *C. serrulatum* to three *Membranoptera* species, followed by *Dasya* and *Calogrossa* species (Figure 1). Within the monophyly of 16 Ceramiales species, all internal nodes were fully resolved. Although its limited taxon sampling in this study, new plastid genome data of *C. serrulatum* may provide valuable information to resolve the phylogenetic relationships within the Ceramiales, the most species-rich order (2712 spp. www.algaebase.com) in Florideophyceae.

## Data Availability

The plastid genome and raw data of *C. serrulatum* is available in the NCBI GenBank under accession number of MW292565 (https://www.ncbi.nlm.nih.gov/nuccore/MW292565) and the NCBI Sequence Read Archive (SRA) under the accession number of SRX9855090 with the BioProject accession number PRJNA692315 (https://www.ncbi.nlm.nih.gov/bioproject/PRJNA692315).
